# Revisiting Mouse Peritoneal Macrophages: Heterogeneity, Development, and Function

**DOI:** 10.3389/fimmu.2015.00225

**Published:** 2015-05-19

**Authors:** Alexandra dos Anjos Cassado, Maria Regina D’Império Lima, Karina Ramalho Bortoluci

**Affiliations:** ^1^Departamento de Imunologia, Instituto de Ciências Biomédicas, Universidade de São Paulo, São Paulo, Brazil; ^2^Centro de Terapia Celular e Molecular (CTC-Mol), Universidade Federal de São Paulo, São Paulo, Brazil; ^3^Departamento de Ciências Biológicas, Campus Diadema, Universidade Federal de São Paulo, São Paulo, Brazil

**Keywords:** peritoneal macrophages, peritoneal cavity, LPM, SPM, origin

## Abstract

Tissue macrophages play a crucial role in the maintenance of tissue homeostasis and also contribute to inflammatory and reparatory responses during pathogenic infection and tissue injury. The high heterogeneity of these macrophages is consistent with their adaptation to distinct tissue environments and specialization to develop niche-specific functions. Although peritoneal macrophages are one of the best-studied macrophage populations, recently it was demonstrated the co-existence of two subsets in mouse peritoneal cavity (PerC), which exhibit distinct phenotypes, functions, and origins. These macrophage subsets have been classified, according to their morphology, as large peritoneal macrophages (LPMs) and small peritoneal macrophages (SPMs). LPMs, the most abundant subset under steady state conditions, express high levels of F4/80 and low levels of class II molecules of the major histocompatibility complex (MHC). LPMs appear to be originated from embryogenic precursors, and their maintenance in PerC is regulated by expression of specific transcription factors and tissue-derived signals. Conversely, SPMs, a minor subset in unstimulated PerC, have a F4/80^low^MHC-II^high^ phenotype and are generated from bone-marrow-derived myeloid precursors. In response to infectious or inflammatory stimuli, the cellular composition of PerC is dramatically altered, where LPMs disappear and SPMs become the prevalent population together with their precursor, the inflammatory monocyte. SPMs appear to be the major source of inflammatory mediators in PerC during infection, whereas LPMs contribute for gut-associated lymphoid tissue-independent and retinoic acid-dependent IgA production by peritoneal B-1 cells. In the previous years, considerable efforts have been made to broaden our understanding of LPM and SPM origin, transcriptional regulation, and functional profile. This review addresses these issues, focusing on the impact of tissue-derived signals and external stimulation in the complex dynamics of peritoneal macrophage populations.

## Introduction

Macrophages are resident cells found in almost all tissues of the body, where they assume specific phenotypes and develop distinct functions. Tissue macrophages are considered as immune sentinels because of their strategic localization and their ability to initiate and modulate immune responses during pathogenic infection or tissue injury and to contribute to the maintenance of tissue homeostasis (1–3). Macrophages were first identified in the late 19th century by Élie Metchnikoff (1845–1916) and designated as large phagocytes (4, 5). Based on their phagocytic activity, macrophages were first classified as cells from the reticuloendothelial system, which also comprised endothelial cells, fibroblasts, spleen and lymphoid reticular cells, Kupffer cells, splenocytes, and monocytes (6). However, because endocytosis performed by endothelial cells is a process that is distinct from phagocytosis, by the late 1960s a new classification system for mononuclear phagocytic cells as cells from “mononuclear phagocytic system” (MPS) was proposed (7). The MPS was defined as a group of phagocytic cells sharing morphological and functional similarities, including pro-monocytes, monocytes, macrophages, dendritic cells (DCs), and their bone marrow (BM) progenitors (7–12). Although the phagocytic cells play similar roles in orchestrating the immune response and maintaining tissue homeostasis (11), they represent cell populations that are extremely heterogeneous (13), and the general classification of mononuclear cells in a unique system is currently under intense discussion (12, 14). In this context, Guilliams et al. suggested a classification of MPS cells based primarily on their ontogeny and secondary on their location, function, and phenotype, promoting a better classification under both steady state and inflammatory conditions (14).

In the last few years, a complex scenario to describe macrophage origins has been developed (15–19), replacing the simplistic view of myeloid precursors giving rise to blood monocytes that, in turn, originate tissue macrophages (20–22). For example, resident macrophages from brain, lung, liver, peritoneum, and spleen are not differentiated from monocytes; instead, they are derived from an embryonic precursor and maintained by self-renewal (23–27). In addition to resident macrophages, infiltrating monocytes are also found in injured tissues, where they can differentiate into inflammatory macrophages or TNF-α- and inducible nitric oxide synthase (iNOS)-producing (Tip)-DCs (28). Currently, it is accepted that inflammatory macrophages and tissue-resident macrophages comprise developmentally and functionally distinct populations (3, 14, 17, 18, 29).

Under steady state conditions, some tissues and serous cavities, including lung, spleen, and the peritoneal cavity (PerC), present distinct resident macrophage subpopulations. In the spleen, at least three macrophage subsets are found: red pulp, metalophilic, and marginal zone macrophages (30). In the PerC, two peritoneal macrophage subsets have been described: large peritoneal macrophage (LPM) and small peritoneal macrophage (SPM) (31). Mouse peritoneal macrophages are among the best-studied macrophage populations in terms of cell biology, development, and inflammatory responses (24, 31–42). Peritoneal macrophages play key roles in the control of infections and inflammatory pathologies (43, 44), as well as in the maintenance of immune response robustness (40). Therefore, this review will discuss recent advances in our understanding of peritoneal macrophage subsets characterization, origin and functions, and the accurate experimental approaches to analyze them.

## Identification of Peritoneal Macrophages

Cohn and collaborators introduced the study of peritoneal macrophages (45–48). Indeed, a representative portion of the current knowledge regarding macrophage biology, such as their function, specialization, and development stems from studies performed using peritoneal macrophages as a cellular source. However, the existence of two resident macrophage subsets present in the PerC was described recently (31). These macrophage subsets were designated LPM and SPM according to their size. LPMs and SPMs were initially identified based on their differential expression of F4/80 and CD11b, where LPMs express high levels of F4/80 and CD11b while SPMs show F4/80^low^CD11b^low^ phenotype (Table 1). CD11b is an integrin that, together with CD18, forms the CR3 heterodimer (13, 30, 49), but is not exclusively expressed on macrophages and is found on several others cell types, including polymorphonuclear cells (50, 51), DCs (52), and at low levels on B lymphocytes (53, 54). F4/80, a 160 kD glycoprotein from the epidermal growth factor (EGF)-transmembrane 7 (TM7) family, is expressed by macrophages in several organs, such as the kidney (55), BM (56), epithelium (57), lung (58, 59), lymphoid organs (60), and among others (61, 62), and it is not found on fibroblasts, polymorphonuclear cells, and lymphocytes (63). However, peritoneal eosinophils show low levels of F4/80 (31) and some macrophage subpopulations exhibit low levels or do not express F4/80, such as white pulp and marginal zone splenic macrophages (30). Therefore, F4/80 expression levels distinguish macrophage subpopulations, including those residing in the same tissue, such as subsets found in the spleen and PerC (30, 31, 35). In this sense, the great majority (approximately 90%) of F4/80^+^CD11b^+^ cells present in the PerC from several mouse strains, including BALB/c, C57BL/6, 129/S6, FVB/N, SJL/J, and RAG^−/−^, express high levels of these molecules and correspond to the LPM subset, whereas the minor SPM subset expresses low levels of these markers (31).

**Table 1:** **Phenotypic profile of SPMs and LPMs**.

Surface molecule	LPMs	SPMs
F4/80	+++	+
CD11b	+++	+
CD11c	+	−
MHC-II	+	++
GR1	+	−
Ly6C	−	−
c-kit	−	−
CD62L	−	++
Dectin-1	+	++
DC-Sign	−	++
TLR4	++	+
CD80	++	+
CD86	+++	+
CD40	++	+
12/15-LOX	+	−
TIM4	+	−

An accurate evaluation of SPMs and LPMs by flow cytometry and optical microscopy revealed that in addition to the differential expression of CD11b and F4/80, SPMs and LPMs display unique morphologies and phenotypes. LPMs assume the classical morphology described for macrophages after adherence, exhibiting prominent vacuolization and abundant cytoplasm, whereas SPMs display a polarized morphology in culture, presenting dendrites similar to DCs (35). Moreover, the analysis of a complex panel of cell surface molecules (Table 1) demonstrated that SPMs express higher levels of MHC-II (IA^b^), dectin-1, and DC-sign endocytic receptors than LPMs. Moreover, half of SPM subset expresses high levels CD62L (31, 35, 36). Conversely, LPMs express higher levels of toll like receptor (TLR)-4 and co-stimulatory molecules in comparison to SPMs (31, 35, 36).

Given that PerC is a singular compartment where specialized immune cells reside and interact, including macrophages, B cells, DCs, eosinophils, mast cells, neutrophils, T cells, natural killer (NK), and invariant NKT cells (31, 32, 35, 36, 64), the identification of myeloid cells from PerC based on cell surface molecules is still a complex matter, particularly in terms of distinguishing macrophage subsets from DCs and inflammatory monocytes. The expression of 12/15-lipoxygenase (LOX), Tim4, and Ly6B has also been examined to discriminate heterogeneous macrophage subsets in PerC under steady state conditions and during peritonitis (24, 37, 38, 42). The high expression of 12/15-LOX and Tim4 was observed in peritoneal macrophages, which also express high levels of F4/80 and CD11b, correlating with the phenotype and frequencies observed for LPMs (24, 31, 37, 38, 42). Conversely, 12/15-LOX^-^ cells and SPM share the same CD11b^+^F4/80^low^MHCII^high^ phenotype; however, 12/15-LOX^-^ cells express high levels of CD11c and co-stimulatory molecules, suggesting that 12/15-LOX^-^ cells and SPMs are, at least in part, distinct populations (31, 35, 37). Despite similarities in cell morphology and MHC-II expression presented by SPMs and DCs, the possibility that SPMs may be part of the peritoneal DC pool is excluded by the smaller size, the distinct and lack of the CD11b and F4/80 expression presented by DCs and, primarily, by the lower expression of CD11c (HL3 or N418 clones of monoclonal anti-CD11c) on SPMs compared with LPMs or typical peritoneal DCs (31, 35).

Given the cell complexity present in PerC and the importance of the development of efficient strategies to correctly identify macrophage subsets as well as to avoid contamination by other cell populations and misinterpretation of peritoneal macrophage studies, our group has proposed a simple way to identify peritoneal macrophage subsets using a four-color flow cytometry staining panel. From doublet, CD19^high^ and CD11c^high^ discarded selected cell populations; the analysis of F4/80^+^ cells based on MHCII expression defines three distinct subpopulations, F4/80^high^IA^b-neg^, F4/80^low^IA^b-high^, and F4/80^low^IA^b-neg^, which correspond, respectively, to LPMs, SPMs, and granulocytes (35).

## Origin and Development of LPM and SPM

The theories that explain the origin of macrophages have been completely reformulated in the last few years. The differentiation process of monocytes, macrophages, and DCs that occurs in the BM starts with the earliest progenitor, the hematopoietic stem cell (HSC), and follows the common myeloid progenitor (CMP) and the granulocyte and macrophage progenitor (GMP) (16). The clonotypic BM-resident precursor differentiated from GMP, termed the macrophage-DC precursor (MDP), expresses high levels of the fractalkine receptor CX3CR1, c-kit, and CD115, and gives rise to circulating blood monocytes, some macrophage populations and a common DC precursor (CDP), but does not originate granulocytes (15, 65, 66). The recruitment of monocyte subsets under steady state or inflammatory and pathological conditions depends on particular chemokines and the expression of their counterpart’s receptors. The Ly6C^+^ monocyte subset migrates via a CCR2-dependent pathway, whereas Ly6C^-^ appears to migrate in response to CX3CR1 signaling (67). Under steady state conditions, extravasated monocytes do not contribute to the pool of resident macrophages in many tissues (3, 15, 16). In inflammatory settings, the Ly6C^+^ monocyte subset differentiates into inflammatory macrophages and monocyte-derived DCs, such as Tip-DCs (15, 16).

Recent accumulating evidence supports the prenatal origin of tissue-resident macrophages and the idea that they are maintained locally by self-renewal throughout adult life, both in the steady state and after cell turnover, which is predominantly independent of hematopoiesis (17, 18, 23–27, 29, 68, 69). Microglia, Langerhans cells, Kupffer cells, red pulp splenic macrophages, lung, and peritoneal macrophages are originated from embryogenic precursor and proliferative cells maintained by self-renewal (23–27, 69–71). Fetal-liver monocytes or primitive macrophages found in the yolk sac, an extraembryonic tissue, have been related with the origin of tissue-resident macrophages. In this context, recent date using yolk sac macrophages depletion and fate-mapping models demonstrated that yolk sac macrophages, which are generated from early erythro-myeloid progenitors (EMPs), are important for development of macrophages in mid-gestation; however in adulthood, only microglia is maintained by these embryogenic precursor (69). In contrast, fetal monocytes that are derived from late EMPs give rise to tissue-resident macrophages from liver, lung, skin, kidney and spleen (69). The exception to the origin of resident macrophages is intestinal macrophages, which are continuously repopulated by circulating monocytes (72).

Understanding the dynamics of maintenance and recruitment of peritoneal macrophages is of particular interest since these cells are involved in physiological as well as pathological processes, such as peritonitis, tumors, and pancreatitis (40, 43, 44). Early studies demonstrated that peritoneal macrophages are maintained in PerC through self-renewal in the steady state or under inflammatory conditions (73–76). The omentum, a fat tissue that connects the abdominal organs, is also involved in peritoneal macrophage development through the proliferative capacities of omental macrophages (75, 76). The combination of these early observations, which were acquired recently, with the technical advances to correctly identify the peritoneal macrophage subsets has permitted the ontogeny of the peritoneal macrophage subsets to be elucidated (24, 31, 36, 39, 40, 42).

**Figure 1:**
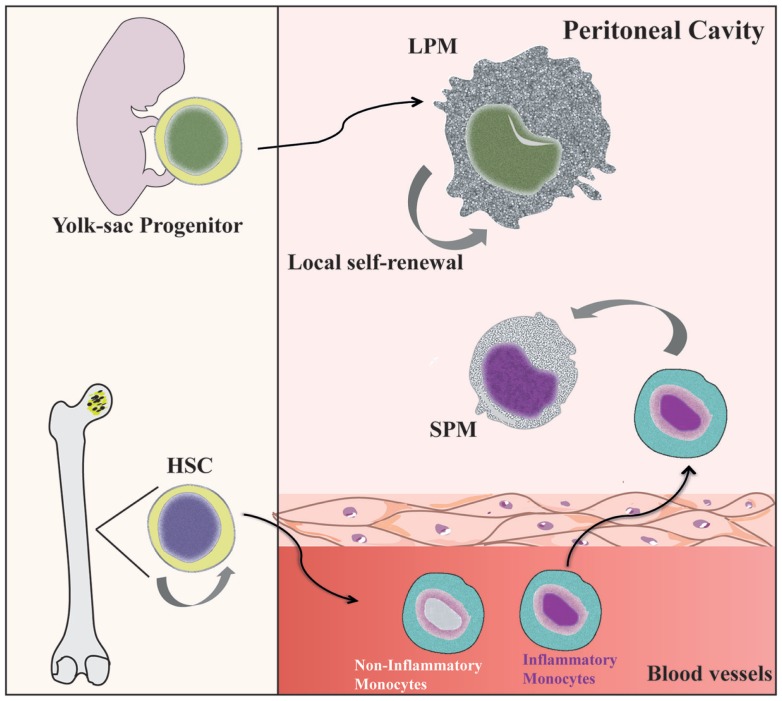
**Distinct origin of peritoneal macrophage subsets**. SPMs are generated from hematopoietic stem cells (HSC) in the bone marrow (BM) by differentiation of inflammatory blood monocytes (31, 40). However, LPMs appear to be originated from progenitors from yolk sac and independent of hematopoietic progenitors (69). Local proliferation of LPMs ensures homeostatic maintenance by self-renewal (36).

Under steady state conditions, LPMs appear to be maintained by self-renewal and independent of hematopoiesis (26, 36), whereas SPMs are originated from circulating monocytes (31, 36, 40) (Figure 1). Dates from Schulz et al. suggest that, in general, F4/80 expression by tissue macrophages correlated with yolk sac (F4/80^high^) and not hematopoietic (F4/80^low^) progenitors (25). In the CX3CR1^GFP/WT^ mice, Cain et al. (36) showed the presence of GFP^+^ cells in DC and SPM pool, but not in the LPM population. Conversely, in the CX_3_CR1CreRosa26R-FGFP mice, which show the active and past expression of CX3CR1, the presence of GFP^+^ cells was found within DC, SPM, and LPM populations. These data indicate that SPMs are short-lived cells, whereas LPMs have a more distant ontogenic relationship with a CX3CR1^+^ progenitor, corroborating the idea that they originate from the yolk sac (36). However, in chimeric C57BL/6 mice reconstituted with C57BL/6-CD45.1 BM, around 80% of SPMs and more than 70% of LPMs are CD45.1-expressing cells, demonstrating that both peritoneal macrophage subsets differentiate from BM precursors after ablation of peritoneal macrophages induced by irradiation (36). Data from our group suggest that PerC recruited Ly6C^+^ monocytes could give rise to SPMs during inflammatory conditions (31). Confirming that SPMs are generated via the differentiation of inflammatory monocytes recruited to PerC, reduced numbers of SPMs are found in the PerC of CCR2^−/−^ mice (40).

The analysis of Ki67 and phosphorylated histone H3 (pHH3 at a discrete stage of mitosis) staining and the quantification of cell cycle and basal DNA content revealed that the number of proliferating F4/80^high^CD11b^high^ cells decreases in 12-week-old mice compared with proliferation capacity of this population in newborn mice (15 days to 4 weeks) (24). After 12–16 weeks, the number of F4/80^high^CD11b^high^cells in PerC is maintained under a low rate of proliferation, which suggests that the number of F4/80^high^CD11b^high^ peritoneal cells increases during mouse development until PerC acquires sufficient homeostatic cell numbers (24). Indeed, BrdU-labeled LPM frequencies after a single BrdU pulse were 7 and 15-fold lower than those found in HSC and GMP, respectively. Moreover, the presence of BrdU^+^ LPMs was detectable 14 days after BrdU pulse, suggesting that they are a long-lived population, i.e., maintained at low levels of proliferation (36). Conversely, the detection of low numbers of proliferating SPMs at 6–10 days after one pulse of BrdU suggests that these cells have a low proliferation rate under steady state conditions and are short-lived cells (36).

Studies with mice deficient in CCAAT/enhancer binding protein (C/EBP)b also support the notion that LPMs and SPMs represent distinct ontogenies, because in the absence of this transcription factor, PerC did not contain LPMs and exhibited increased numbers of SPMs (36). Interestingly, adoptively transferred SPMs differentiated into LPMs in Cebpb^−/−^ mice. However, in control mice that have normal numbers of LPMs, only a small frequency of transferred SPMs acquired the F4/80^hi^MHCII^low^CD93^+^ phenotype of LPMs. Based on these results, the authors proposed that under physiological conditions, SPMs appear to contribute in only a small way to generate LPMs, but SPMs could be involved in the maintenance of LPMs in situations where this pool has been greatly reduced, such as under inflammatory conditions or following radiation ablation (36). These data are consistent with the findings of Yona et al. (26), which demonstrated the presence of monocyte-derived cells in the LPM compartment 8 weeks after the i.p. injection of thioglycollate. Together with LPMs, a subset of proliferating BM-derived inflammatory macrophage has also been associated with self-renewal mechanisms during the resolution of peritonitis induced by zymosan and thioglycollate (42). Conversely, LPMs do not seem to contribute to the SPM pool, even during inflammation. Our group demonstrated that adoptively transferred CFDA-SE-labeled LPMs 1 h after LPS stimulation retained its phenotype, and no CFDA-SE^+^ cells were found in the SPM compartment until 2 days after stimulation (31).

In the last year, a great advance in the understanding of the transcriptional control of peritoneal macrophages provided novel insights into this scenario (39, 40). The zinc finger transcription factor GATA-binding protein 6 (GATA6) appears to regulate the majority of peritoneal macrophage-specific genes (PMSGs). Of note, GATA6 is selectively expressed by LPMs (40). Accordingly, the number of LPMs were greatly reduced in peritoneal lavages from GATA6-KO^mye^ and Mac-GATA6 KO mice, which have a GATA6 deficiency in all myeloid cells or only in the macrophage lineages, respectively (39, 40). Interestingly, retinoic acid (RA) is the extracellular factor that regulates GATA-6-specific gene expression in LPMs, because vitamin A depleted (VAD; the RA precursor) mice exhibited a decrease in GATA6 expression and LPM numbers (40). Moreover, the stimulation of peritoneal macrophages from VAD mice with all-trans RA restored the expression of GATA-6 and many PMSGs at levels found in peritoneal macrophages from control mice. In addition to the regulation of gene expression profiling in peritoneal macrophages, GATA-6 appears to be involved in the control of the proliferation, survival, and metabolism of these cells (39, 77). GATA-6-deficient macrophages demonstrate an altered proliferation state during peritonitis (39). Moreover, Lyz2-Cre × GATA6^(flox/flox)^ mice also exhibit reduced numbers of peritoneal macrophages, which could be explained by the perturbation in their metabolism, culminating in the high frequency of cell death found in this compartment (77). Despite great contributions to our understanding in the involvement of GATA-6 in peritoneal macrophage development, metabolism, self-maintenance, and survival, the existence of distinct pathways that could govern the transcriptional regulation of SPMs remains largely unknown.

In addition to transcriptional regulation, signaling factors derived from the microenvironment also play an essential role in promoting the development and phenotype of tissue-resident macrophages. For example, TGF-β1 signaling is required for the development of the microglia population and to regulate a microglia expression program through the Smad tissue factors (78–80). Heme has been shown to induce Spi-c, a transcription factor important for red pulp macrophage development (81, 82). Finally, in PerC, omentum-derived RA promotes the expression of GATA-6 in the LPM subset, determining its localization and functions (40), even if the factors that maintain the SPM pool under steady state conditions still remain to be elucidated.

## Dynamics and Function of Peritoneal Macrophage Subsets

**Figure 2:**
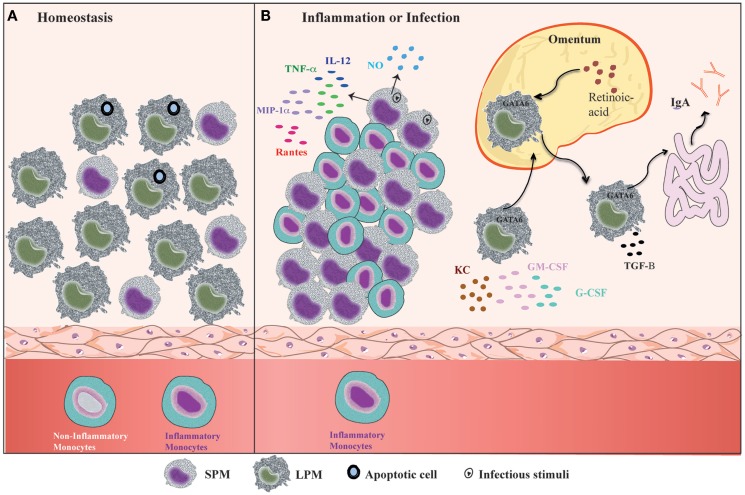
**Summary of the dynamic of peritoneal macrophage subsets**. **(A)** Under homeostatic conditions, peritoneal macrophages comprise two subsets LPMs and SPMs (31). LPMs, which are the major peritoneal macrophage population, appear to be responsible for phagocytosis of apoptotic cell and tissue repair (36). **(B)** At the outset of inflammation, the myeloid compartment is modified in general by disappearance of LPMs, increase of SPMs numbers, and monocytes influx (31, 35, 36, 40). The changes in the myeloid cells from zymosan, *T. cruzi*, and LPS stimulated or thioglicollate-elicited PerC result in the gain of immune state (35, 36). SPMs from zymosan and *T. cruzi* stimulated mice contribute to effector function of PerC through secretion of high levels of NO and presence of IL-12-producing cells (35). In response to LPS *in vivo*, SPMs produce several inflammatory cytokines, such as IL-12, MIP-1α, TNF-α, and RANTES, whereas LPMs produce enhanced amounts of G-CSF, GM-CSF, and KC (36). LPMs, which migrate to omentum by a retinoic acid and GATA-6-dependent way in response to *in vivo* LPS stimulation or vitamin-A deprivation, return to PerC and appear to be correlated with GALT-independent and TGF-β2-dependent IgA production by B-1 cells in the intestine (40).

Mouse PerC is a compartment where many cell types co-habitat and interact, similar to the secondary lymphoid organs. In addition, PerC is a unique body compartment that contains B-1 cells (83). Under steady state conditions, the peritoneal cells comprise LPMs, SPMs, B-1 cells, conventional B-2 cells, T cells, NK cells, DCs, and granulocytes (mostly eosinophils) (31, 35). B1 cells constitute the majority of the PerC cell population, whereas the SPM and LPM frequencies represent 30–35% of total peritoneal cells (31, 35) (Figure 2A). However, after inflammatory or infectious stimuli, there is a dramatic alteration in cell numbers and the frequencies of each of PerC cell subpopulation. With regard to the myeloid compartment, modifications in PerC cell composition include the disappearance of LPMs, increases in SPM frequency and numbers, and a massive recruitment of inflammatory monocytes (24, 31, 35, 36, 40) (Figure 2B).

The “macrophage disappearance reaction” (MDR) in PerC has been extensively described during delayed-type hypersensitivity (DTH) and acute inflammatory processes (84). MDR has been associated with cell death, emigration to draining lymph nodes, or adherence of macrophages to structural tissues. LPMs are the unique peritoneal macrophage subset that disappears from PerC, which is attributed not to cell death but rather to their migration to the omentum (31, 40). LPM disappearance in response to inflammatory stimuli is accompanied by an increase in SPM and inflammatory monocyte numbers (24, 31, 35, 36, 40) (Figure 2B), and has been correlated with the renewal and improvement of immune conditions of the PerC (35). Adherent peritoneal cells from naive mice, which are composed primarily of LPM, exhibit a high frequency of cells stained for β-galactosamine (β-gal), a senescence marker (85–87). These cells are unable to secrete NO in response to LPS challenge (35). In contrast, adherent peritoneal cells from *Trypanosoma cruzi* or zymosan-stimulated mice in which the main cell population constitutes SPMs and monocytes (F4/80^low^MHCII^int^Ly-6C^+^), respectively, display a significant reduction in the frequency of β-gal-positive cells and secrete high levels of NO in response to LPS (35). The frequency of IL-12-producing cells after *in vitro* LPS plus IFN-γ stimulation was also higher within myelo-monocytic cells from mice exposed to zymosan and *T. cruzi* than the frequencies of IL-12-producing cells found in unstimulated mice (35). In response to *Staphylococcus epidermidis* cell-free (SES) supernatant *in vivo* stimulation, F4/80^low^CD11b^+^ cells (consisting of SPMs and DCs) produced enhanced levels of IL-1β, IL-1α, TNF-α, and IL-12 in the presence or absence of subsequent SES treatment (37). In contrast, the supernatants of adherent cells from naïve mice treated with SES were found to contain high levels of MCP-1, MCP-1α, MIP-1β, and G-CSF (37). It is important to note that 4 days after thioglycollate injection, peritoneal cells, an extensively studied cell population (88–91), also consist primarily of SPMs and inflammatory monocytes (31, 40). The increase in SPM numbers and the influx of inflammatory monocytes that will give rise to SPMs greatly contribute to the improvement of the capacity of PerC to deal with inflammatory stimuli. Indeed, although neither SPMs nor LPMs produce significant levels of pro- or anti-inflammatory cytokines under steady state conditions (35–37), SPMs appear to develop a pro-inflammatory profile in response to *in vitro* stimuli. SPMs produced high levels of TNF-α, MIP-1α, and RANTES in response to LPS, whereas LPMs were the unique population that produced abundant levels of G-CSF, GM-CSF, and KC in response to the same stimulus (36) (Figure 2B).

The NO secretion and pro-inflammatory cytokine production are the most important functions of activated macrophages by inflammatory stimulation and assigns the M1 profile (13, 34, 92–97). The functional profile of peritoneal macrophages was previous studied by our group and others (33, 34). Peritoneal macrophages from Th1-prone mouse strains (C57BL/6 and B10.A) are easily activated to produce NO in response to rIFN-γ or LPS, characterizing the M1 profile. In contrast, macrophages from Th2-prone mouse strains (BALB/c and DBA/2) exhibit a weak NO response as a consequence of high levels of spontaneously secreted TGF-β1 (34). Moreover, the cells from C57BL/6 IL-12p40-deficient mice have a bias toward the M2 profile, indicating that IL-12 is required for M1 polarization of peritoneal macrophages (33). Although LPMs from naïve mice can produce NO after *in vitro* LPS stimulation, SPMs produce higher levels of NO than LPMs following *in vivo* LPS stimulation. The NO secretion by LPMs was also detected by flow cytometry in *Escherichia coli* inoculated mice (31), whereas nitrite was not produced *in vitro* by LPS-stimulated adherent peritoneal cells from control mice, which is composed mainly by LPMs (35). In addition, adherent cells obtained 48 h after *T. cruzi* infection, which are mostly composed by SPMs, were the unique source of NO without *in vitro* subsequent challenge with LPS (35). In resume, the SPM and LPM subsets cannot be accommodated in the M1/M2 framework considering the NO secretion. However, considering phagocytic assays, SPMs appear to develop an efficient profile to control infections as M1 macrophages, whereas LPMs assume a role in the maintenance of PerC physiological conditions as M2 or alternative macrophages. Despite the preserved phagocytic ability of LPMs, higher numbers of zymosan and *E. coli* were found inside of SPMs at early time points after i.p. injection (31, 35). Conversely, at 1 h after challenge, LPMs appear to present a higher phagocytic index of apoptotic thymocytes in comparison to SPMs (36) (Figure 2A).

In addition, it was recently demonstrated that LPMs have a unique ability to induce gut-associated lymphoid tissue (GALT)-independent IgA production by peritoneal B-1 cells (40) (Figure 2B). RA and TGF-β2 are the most critical factors to induce IgA class switching, and the production of TGF-β2 is regulated by the *Tgfb2* and *Ltbp1* genes, which are expressed by LPMs in a GATA-6-dependent manner. This process is regulated by the abundant presence of RA in the omentum, which is responsible for the induction of GATA-6 expression in LPMs that migrates to this tissue. The dynamic of LPM migration between the PerC and the omentum after the stimulation of PerC is correlated with their disappearance and the return to basal numbers of LPMs later after stimulation with LPS, zymosan, and thioglycollate (24, 31, 35, 36, 39, 40). This observation suggests that LPMs can return to PerC to resolve an infectious or inflammatory process. Therefore, the presence of two specialized macrophage subsets in PerC is crucial to maintain the health of this compartment under different situations.

## Concluding Remarks

Peritoneal macrophages represent one of the most studied macrophage populations. However, the existence of two phenotypically and functionally distinct subsets, LPMs and SPMs, residing in the PerC was recognized recently (31). In the last year, great advances in our understanding of the transcriptional regulation of peritoneal macrophages have brought novel insights into the identification of LPMs and SPMs (39, 40). GATA-6, an LPM-restricted transcription factor, regulates many PMSGs, including those related to the maintenance of LPMs in PerC (40) and those that determine their function (40), metabolism, proliferation, and cell survival (39, 77). Under steady state conditions, LPMs appear to originate independently from hematopoietic precursors and retained the ability to proliferate *in situ*, maintaining physiological numbers (26, 36). Conversely, SPMs appear to originate from circulating monocytes (31, 36, 40), and their numbers increase remarkably under inflammatory conditions. Of note, SPMs together with their precursor, the inflammatory monocyte population, are the major myeloid populations present in elicited PerC, and are an excellent resource to study the biology of inflammatory macrophages. SPMs and LPMs exhibit specialized functions in the PerC, where SPMs present a pro-inflammatory functional profile, and LPMs appear to have a role in the maintenance of PerC physiological conditions. Moreover, the particular interactions between macrophage subsets and other peritoneal cell populations appear to play crucial roles in PerC immune state. Although the consequences of the crosstalk between SPMs and peritoneal T and B lymphocytes remain to be clarified, LPMs are required for GALT-independent and RA-dependent IgA production by peritoneal B-1 cells (40). Finally, the elucidation of the influence of soluble factors and the microbiota on the maintenance of LPM/SPM ratios in PerC, and the role of these subsets in the systemic immune response are the future challenges for this field.

## Conflict of Interest Statement

The authors declare that the research was conducted in the absence of any commercial or financial relationships that could be construed as a potential conflict of interest.

## References

[1] Taylor PR Gordon S Monocyte heterogeneity and innate immunity. Immunity (2003) 19(1):2–4. 10.1016/S1074-7613(03)00178-X 12871633

[2] Gordon S. The macrophage: past, present and future. Eur J Immunol (2007) 37(Suppl 1):S9–17. 10.1002/eji.200737638 17972350

[3] Wynn TA Chawla A Pollard JW Macrophage biology in development, homeostasis and disease. Nature (2013) 496(7446):445–55. 10.1038/nature12034 23619691 PMC3725458

[4] Metchnikoff E Leçons sur la Pathologie Comparée de I’inflammation faites à l’Institut Pasteur en 1891. Paris: Masson (1892).

[5] Van M [Elie Metchnikoff, 1845-1916]. Voeding (1964) 25:351–6. 14260770

[6] Aschoff L Das reticuloendotheliale system. Ergeb Inn Med Kinderheilkd (1924) 26:1–117.

[7] van Furth R Cohn ZA Hirsch JG Humphrey JH Spector WG Langevoort HL. The mononuclear phagocyte system: a new classification of macrophages, monocytes, and their precursor cells. Bull World Health Organ (1972) 46(6):845–52. 4538544 PMC2480884

[8] van Furth R. Current view of the mononuclear phagocyte system. Haematol Blood Transfus (1981) 27:3–10. 7327435 10.1007/978-3-642-81696-3_1

[9] van Furth R The mononuclear phagocyte system. Verh Dtsch Ges Pathol (1980) 64:1–11. 7013331 10.1007/978-94-009-8793-7_1

[10] Hume DA Ross IL Himes SR Sasmono RT Wells CA Ravasi T. The mononuclear phagocyte system revisited. J Leukoc Biol (2002) 72(4):621–7. 12377929

[11] Hume DA The mononuclear phagocyte system. Curr Opin Immunol (2006) 18(1):49–53. 10.1016/j.coi.2005.11.008 16338128

[12] Geissmann F Gordon S Hume DA Mowat AM Randolph GJ. Unravelling mononuclear phagocyte heterogeneity. Nat Rev Immunol (2010) 10(6):453–60. 10.1038/nri2784 20467425 PMC3032581

[13] Gordon S Taylor PR Monocyte and macrophage heterogeneity. Nat Rev Immunol (2005) 5(12):953–64. 10.1038/nri1733 16322748

[14] Guilliams M Ginhoux F Jakubzick C Naik SH Onai N Schraml BU Dendritic cells, monocytes and macrophages: a unified nomenclature based on ontogeny. Nat Rev Immunol (2014) 14(8):571–8. 10.1038/nri3712 25033907 PMC4638219

[15] Auffray C Sieweke MH Geissmann F. Blood monocytes: development, heterogeneity, and relationship with dendritic cells. Annu Rev Immunol (2009) 27:669–92. 10.1146/annurev.immunol.021908.132557 19132917

[16] Geissmann F Manz MG Jung S Sieweke MH Merad M Ley K. Development of monocytes, macrophages, and dendritic cells. Science (2010) 327(5966):656–61. 10.1126/science.1178331 20133564 PMC2887389

[17] Davies LC Jenkins SJ Allen JE Taylor PR. Tissue-resident macrophages. Nat Immunol (2013) 14:986–95. 10.1038/ni.2705 24048120 PMC4045180

[18] Ginhoux F Jung S Monocytes and macrophages: developmental pathways and tissue homeostasis. Nat Rev Immunol (2014) 14(6):392–404. 10.1038/nri3671 24854589

[19] Ginhoux F Merad M. Ontogeny and homeostasis of Langerhans cells. Immunol Cell Biol (2010) 88(4):387–92. 10.1038/icb.2010.38 20309014

[20] van Furth R [The origin of mononuclear phagocytes]. Ned Tijdschr Geneeskd (1967) 111(48):2208. 5584286

[21] van Furth R Cohn ZA The origin and kinetics of mononuclear phagocytes. J Exp Med (1968) 128(3):415–35. 10.1084/jem.128.3.415 5666958 PMC2138527

[22] van Furth R Origin and kinetics of monocytes and macrophages. Semin Hematol (1970) 7(2):125–41. 4986254

[23] Ginhoux F Greter M Leboeuf M Nandi S See P Gokhan S Fate mapping analysis reveals that adult microglia derive from primitive macrophages. Science (2010) 330(6005):841–5. 10.1126/science.1194637 20966214 PMC3719181

[24] Davies LC Rosas M Smith PJ Fraser DJ Jones SA Taylor PR. A quantifiable proliferative burst of tissue macrophages restores homeostatic macrophage populations after acute inflammation. Eur J Immunol (2011) 41(8):2155–64. 10.1002/eji.201141817 21710478

[25] Schulz C Gomez Perdiguero E Chorro L Szabo-Rogers H Cagnard N Kierdorf K A lineage of myeloid cells independent of Myb and hematopoietic stem cells. Science (2012) 336(6077):86–90. 10.1126/science.1219179 22442384

[26] Yona S Kim KW Wolf Y Mildner A Varol D Breker M Fate mapping reveals origins and dynamics of monocytes and tissue macrophages under homeostasis. Immunity (2013) 38(1):79–91. 10.1016/j.immuni.2012.12.001 23273845 PMC3908543

[27] Hashimoto D Chow A Noizat C Teo P Beasley MB Leboeuf M Tissue-resident macrophages self-maintain locally throughout adult life with minimal contribution from circulating monocytes. Immunity (2013) 38(4):792–804. 10.1016/j.immuni.2013.04.004 23601688 PMC3853406

[28] Auffray C Fogg DK Narni-Mancinelli E Senechal B Trouillet C Saederup N CX3CR1+ CD115+ CD135+ common macrophage/DC precursors and the role of CX3CR1 in their response to inflammation. J Exp Med (2009) 206(3):595–606. 10.1084/jem.20081385 19273628 PMC2699130

[29] Sieweke MH Allen JE. Beyond stem cells: self-renewal of differentiated macrophages. Science (2013) 342(6161):1242974. 10.1126/science.1242974 24264994

[30] Taylor PR Martinez-Pomares L Stacey M Lin HH Brown GD Gordon S Macrophage receptors and immune recognition. Annu Rev Immunol (2005) 23:901–44. 10.1146/annurev.immunol.23.021704.115816 15771589

[31] Ghosn EE Cassado AA Govoni GR Fukuhara T Yang Y Monack DM Two physically, functionally, and developmentally distinct peritoneal macrophage subsets. Proc Natl Acad Sci U S A (2010) 107(6):2568–73. 10.1073/pnas.0915000107 20133793 PMC2823920

[32] Schleicher U Hesse A Bogdan C. Minute numbers of contaminant CD8+ T cells or CD11b+CD11c+ NK cells are the source of IFN-gamma in IL-12/IL-18-stimulated mouse macrophage populations. Blood (2005) 105(3):1319–28. 10.1182/blood-2004-05-1749 15383459

[33] Bastos KR Alvarez JM Marinho CR Rizzo LV Lima MR. Macrophages from IL-12p40-deficient mice have a bias toward the M2 activation profile. J Leukoc Biol (2002) 71(2):271–8. 11818448

[34] Mills CD Kincaid K Alt JM Heilman MJ Hill AM. M-1/M-2 macrophages and the Th1/Th2 paradigm. J Immunol (2000) 164(12):6166–73. 10.4049/jimmunol.164.12.6166 10843666

[35] Cassado Ados A de Albuquerque JA Sardinha LR Buzzo Cde L Faustino L Nascimento R Cellular renewal and improvement of local cell effector activity in peritoneal cavity in response to infectious stimuli. PLoS One (2011) 6(7):e22141. 10.1371/journal.pone.0022141 21799778 PMC3142143

[36] Cain DW O’Koren EG Kan MJ Womble M Sempowski GD Hopper K Identification of a tissue-specific, C/EBPbeta-dependent pathway of differentiation for murine peritoneal macrophages. J Immunol (2013) 191(9):4665–75. 10.4049/jimmunol.1300581 24078688 PMC3808250

[37] Dioszeghy V Rosas M Maskrey BH Colmont C Topley N Chaitidis P 12/15-Lipoxygenase regulates the inflammatory response to bacterial products in vivo. J Immunol (2008) 181(9):6514–24. 10.4049/jimmunol.181.9.6514 18941242

[38] Rosas M Thomas B Stacey M Gordon S Taylor PR. The myeloid 7/4-antigen defines recently generated inflammatory macrophages and is synonymous with Ly-6B. J Leukoc Biol (2010) 88(1):169–80. 10.1189/jlb.0809548 20400676 PMC2892525

[39] Rosas M Davies LC Giles PJ Liao CT Kharfan B Stone TC The transcription factor Gata6 links tissue macrophage phenotype and proliferative renewal. Science (2014) 344(6184):645–8. 10.1126/science.1251414 24762537 PMC4185421

[40] Okabe Y Medzhitov R. Tissue-specific signals control reversible program of localization and functional polarization of macrophages. Cell (2014) 157(4):832–44. 10.1016/j.cell.2014.04.016 24792964 PMC4137874

[41] Wang C Yu X Cao Q Wang Y Zheng G Tan TK Characterization of murine macrophages from bone marrow, spleen and peritoneum. BMC Immunol (2013) 14:6. 10.1186/1471-2172-14-6 23384230 PMC3574850

[42] Davies LC Rosas M Jenkins SJ Liao CT Scurr MJ Brombacher F Distinct bone marrow-derived and tissue-resident macrophage lineages proliferate at key stages during inflammation. Nat Commun (2013) 4:1886. 10.1038/ncomms2877 23695680 PMC3842019

[43] Dahdah A Gautier G Attout T Fiore F Lebourdais E Msallam R Mast cells aggravate sepsis by inhibiting peritoneal macrophage phagocytosis. J Clin Invest (2014) 124(10):4577–89. 10.1172/JCI75212 25180604 PMC4191002

[44] Machado MCC Coelho AMM Role of Peritoneal Macrophages on Local And Systemic Inflammatory Response in Acute Pancreatitis. São Paulo: InTech (2012). 10.5772/25639

[45] Cohn ZA Determinants of infection in the peritoneal cavity. I. Response to and fate of *Staphylococcus aureus* and *Staphylococcus albus* in the mouse. Yale J Biol Med (1962) 35:12–28. 13880374 PMC2604499

[46] Cohn ZA Determinants of infection in the peritoneal cavity. II. Factors influencing the fate of *Staphylococcus aureus* in the mouse. Yale J Biol Med (1962) 35:29–47. 13880375 PMC2604494

[47] Cohn ZA Determinants of infection in the peritoneal cavity. III. The action of selected inhibitors on the fate of *Staphylococcus aureus* in the mouse. Yale J Biol Med (1962) 35:48–61. 13880376 PMC2604491

[48] Steinman RM Moberg CL. Zanvil Alexander Cohn 1926-1993. J Exp Med (1994) 179(1):1–30. 10.1084/jem.179.1.1 8270858 PMC2191311

[49] Taylor PR Brown GD Geldhof AB Martinez-Pomares L Gordon S. Pattern recognition receptors and differentiation antigens define murine myeloid cell heterogeneity ex vivo. Eur J Immunol (2003) 33(8):2090–7. 10.1002/eji.200324003 12884282

[50] Hickstein DD Ozols J Williams SA Baenziger JU Locksley RM Roth GJ. Isolation and characterization of the receptor on human neutrophils that mediates cellular adherence. J Biol Chem (1987) 262(12):5576–80. 3553180

[51] Petty HR Todd RF III. Receptor-receptor interactions of complement receptor type 3 in neutrophil membranes. J Leukoc Biol (1993) 54(5):492–4. 8228627 10.1002/jlb.54.5.492

[52] Shortman K Liu YJ. Mouse and human dendritic cell subtypes. Nat Rev Immunol (2002) 2(3):151–61. 10.1038/nri746 11913066

[53] Kantor AB Stall AM Adams S Herzenberg LA. Differential development of progenitor activity for three B-cell lineages. Proc Natl Acad Sci U S A (1992) 89(8):3320–4. 10.1073/pnas.89.8.3320 1565622 PMC48858

[54] Ghosn EE Yang Y Tung J Herzenberg LA. CD11b expression distinguishes sequential stages of peritoneal B-1 development. Proc Natl Acad Sci U S A (2008) 105(13):5195–200. 10.1073/pnas.0712350105 18375763 PMC2278228

[55] Hume DA Gordon S. Mononuclear phagocyte system of the mouse defined by immunohistochemical localization of antigen F4/80. Identification of resident macrophages in renal medullary and cortical interstitium and the juxtaglomerular complex. J Exp Med (1983) 157(5):1704–9. 10.1084/jem.157.5.1704 6854206 PMC2186998

[56] Hume DA Loutit JF Gordon S. The mononuclear phagocyte system of the mouse defined by immunohistochemical localization of antigen F4/80: macrophages of bone and associated connective tissue. J Cell Sci (1984) 66:189–94. 6378941 10.1242/jcs.66.1.189

[57] Hume DA Perry VH Gordon S. The mononuclear phagocyte system of the mouse defined by immunohistochemical localisation of antigen F4/80: macrophages associated with epithelia. Anat Rec (1984) 210(3):503–12. 10.1002/ar.1092100311 6524692

[58] Bedoret D Wallemacq H Marichal T Desmet C Quesada Calvo F Henry E Lung interstitial macrophages alter dendritic cell functions to prevent airway allergy in mice. J Clin Invest (2009) 119(12):3723–38. 10.1172/JCI39717 19907079 PMC2786798

[59] Guilliams M De Kleer I Henri S Post S Vanhoutte L De Prijck S Alveolar macrophages develop from fetal monocytes that differentiate into long-lived cells in the first week of life via GM-CSF. J Exp Med (2013) 210(10):1977–92. 10.1084/jem.20131199 24043763 PMC3782041

[60] Hume DA Robinson AP MacPherson GG Gordon S. The mononuclear phagocyte system of the mouse defined by immunohistochemical localization of antigen F4/80. Relationship between macrophages, Langerhans cells, reticular cells, and dendritic cells in lymphoid and hematopoietic organs. J Exp Med (1983) 158(5):1522–36. 10.1084/jem.158.5.1522 6355361 PMC2187139

[61] Hume DA Perry VH Gordon S. Immunohistochemical localization of a macrophage-specific antigen in developing mouse retina: phagocytosis of dying neurons and differentiation of microglial cells to form a regular array in the plexiform layers. J Cell Biol (1983) 97(1):253–7. 10.1083/jcb.97.1.253 6345555 PMC2112503

[62] Hume DA Halpin D Charlton H Gordon S. The mononuclear phagocyte system of the mouse defined by immunohistochemical localization of antigen F4/80: macrophages of endocrine organs. Proc Natl Acad Sci U S A (1984) 81(13):4174–7. 10.1073/pnas.81.13.4174 6377311 PMC345391

[63] Austyn JM Gordon S. F4/80, a monoclonal antibody directed specifically against the mouse macrophage. Eur J Immunol (1981) 11(10):805–15. 10.1002/eji.1830111013 7308288

[64] Ghosn EE Yang Y Tung J Herzenberg LA Herzenberg LA. CD11b expression distinguishes sequential stages of peritoneal B-1 development. Proc Natl Acad Sci U S A (2008) 105(13):5195–200. 10.1073/pnas.0712350105 18375763 PMC2278228

[65] Fogg DK Sibon C Miled C Jung S Aucouturier P Littman DR A clonogenic bone marrow progenitor specific for macrophages and dendritic cells. Science (2006) 311(5757):83–7. 10.1126/science.1117729 16322423

[66] Landsman L Varol C Jung S. Distinct differentiation potential of blood monocyte subsets in the lung. J Immunol (2007) 178(4):2000–7. 10.4049/jimmunol.178.4.2000 17277103

[67] Geissmann F Jung S Littman DR. Blood monocytes consist of two principal subsets with distinct migratory properties. Immunity (2003) 19(1):71–82. 10.1016/S1074-7613(03)00174-2 12871640

[68] Chorro L Geissmann F. Development and homeostasis of ‘resident’ myeloid cells: the case of the Langerhans cell. Trends Immunol (2010) 31(12):438–45. 10.1016/j.it.2010.09.003 21030305

[69] Hoeffel G Chen J Lavin Y Low D Almeida FF See P C-myb(+) erythro-myeloid progenitor-derived fetal monocytes give rise to adult tissue-resident macrophages. Immunity (2015) 42(4):665–78. 10.1016/j.immuni.2015.03.011 25902481 PMC4545768

[70] Ajami B Bennett JL Krieger C Tetzlaff W Rossi FM. Local self-renewal can sustain CNS microglia maintenance and function throughout adult life. Nat Neurosci (2007) 10(12):1538–43. 10.1038/nn2014 18026097

[71] Chorro L Sarde A Li M Woollard KJ Chambon P Malissen B Langerhans cell (LC) proliferation mediates neonatal development, homeostasis, and inflammation-associated expansion of the epidermal LC network. J Exp Med (2009) 206(13):3089–100. 10.1084/jem.20091586 19995948 PMC2806478

[72] Zigmond E Jung S. Intestinal macrophages: well educated exceptions from the rule. Trends Immunol (2013) 34(4):162–8. 10.1016/j.it.2013.02.001 23477922

[73] Parwaresch MR Wacker HH. Origin and kinetics of resident tissue macrophages. Parabiosis studies with radiolabelled leucocytes. Cell Tissue Kinet (1984) 17(1):25–39. 6692464 10.1111/j.1365-2184.1984.tb00565.x

[74] Melnicoff MJ Horan PK Breslin EW Morahan PS Maintenance of peritoneal macrophages in the steady state. J Leukoc Biol (1988) 44(5):367–75. 2460572 10.1002/jlb.44.5.367

[75] Daems WT de Bakker JM Do resident macrophages proliferate? Immunobiology (1982) 161(3–4):204–11. 10.1016/S0171-2985(82)80075-2 7047370

[76] Wijffels JF Hendrickx RJ Steenbergen JJ Eestermans IL Beelen RH. Milky spots in the mouse omentum may play an important role in the origin of peritoneal macrophages. Res Immunol (1992) 143(4):401–9. 10.1016/S0923-2494(05)80072-0 1518954

[77] Gautier EL Ivanov S Williams JW Huang SC Marcelin G Fairfax K Gata6 regulates aspartoacylase expression in resident peritoneal macrophages and controls their survival. J Exp Med (2014) 211(8):1525–31. 10.1084/jem.20140570 25024137 PMC4113942

[78] Makwana M Jones LL Cuthill D Heuer H Bohatschek M Hristova M Endogenous transforming growth factor beta 1 suppresses inflammation and promotes survival in adult CNS. J Neurosci (2007) 27(42):11201–13. 10.1523/JNEUROSCI.2255-07.2007 17942715 PMC6673043

[79] Abutbul S Shapiro J Szaingurten-Solodkin I Levy N Carmy Y Baron R TGF-beta signaling through SMAD2/3 induces the quiescent microglial phenotype within the CNS environment. Glia (2012) 60(7):1160–71. 10.1002/glia.22343 22511296

[80] Butovsky O Jedrychowski MP Moore CS Cialic R Lanser AJ Gabriely G Identification of a unique TGF-beta-dependent molecular and functional signature in microglia. Nat Neurosci (2014) 17(1):131–43. 10.1038/nn.3599 24316888 PMC4066672

[81] Kohyama M Ise W Edelson BT Wilker PR Hildner K Mejia C Role for Spi-C in the development of red pulp macrophages and splenic iron homeostasis. Nature (2009) 457(7227):318–21. 10.1038/nature07472 19037245 PMC2756102

[82] Haldar M Kohyama M So AY Kc W Wu X Briseno CG Heme-mediated SPI-C induction promotes monocyte differentiation into iron-recycling macrophages. Cell (2014) 156(6):1223–34. 10.1016/j.cell.2014.01.069 24630724 PMC4010949

[83] Baumgarth N. The double life of a B-1 cell: self-reactivity selects for protective effector functions. Nat Rev Immunol (2011) 11(1):34–46. 10.1038/nri2901 21151033

[84] Barth MW Hendrzak JA Melnicoff MJ Morahan PS Review of the macrophage disappearance reaction. J Leukoc Biol (1995) 57(3):361–7. 7884305 10.1002/jlb.57.3.361

[85] Lloberas J Celada A. Effect of aging on macrophage function. Exp Gerontol (2002) 37(12):1325–31. 10.1016/S0531-5565(02)00125-0 12559402

[86] Herrero C Sebastian C Marques L Comalada M Xaus J Valledor AF Immunosenescence of macrophages: reduced MHC class II gene expression. Exp Gerontol (2002) 37(2–3):389–94. 10.1016/S0531-5565(01)00205-4 11772525

[87] Dimri GP Lee X Basile G Acosta M Scott G Roskelley C A biomarker that identifies senescent human cells in culture and in aging skin in vivo. Proc Natl Acad Sci U S A (1995) 92(20):9363–7. 10.1073/pnas.92.20.9363 7568133 PMC40985

[88] Takahashi M Galligan C Tessarollo L Yoshimura T. Monocyte chemoattractant protein-1 (MCP-1), not MCP-3, is the primary chemokine required for monocyte recruitment in mouse peritonitis induced with thioglycollate or zymosan A. J Immunol (2009) 183(5):3463–71. 10.4049/jimmunol.0802812 19641140 PMC7371094

[89] Cohn ZA Activation of mononuclear phagocytes: fact, fancy, and future. J Immunol (1978) 121(3):813–6. 357655

[90] Leijh PC van Zwet TL ter Kuile MN van Furth R. Effect of thioglycolate on phagocytic and microbicidal activities of peritoneal macrophages. Infect Immun (1984) 46(2):448–52. 6500699 10.1128/iai.46.2.448-452.1984 PMC261553

[91] Lagasse E Weissman IL. Flow cytometric identification of murine neutrophils and monocytes. J Immunol Methods (1996) 197(1–2):139–50. 10.1016/0022-1759(96)00138-X 8890901

[92] Mantovani A Sozzani S Locati M Allavena P Sica A. Macrophage polarization: tumor-associated macrophages as a paradigm for polarized M2 mononuclear phagocytes. Trends Immunol (2002) 23(11):549–55. 10.1016/S1471-4906(02)02302-5 12401408

[93] Gordon S Alternative activation of macrophages. Nat Rev Immunol (2003) 3(1):23–35. 10.1038/nri978 12511873

[94] Mantovani A Sica A Sozzani S Allavena P Vecchi A Locati M. The chemokine system in diverse forms of macrophage activation and polarization. Trends Immunol (2004) 25(12):677–86. 10.1016/j.it.2004.09.015 15530839

[95] Martinez FO Helming L Gordon S. Alternative activation of macrophages: an immunologic functional perspective. Annu Rev Immunol (2009) 27:451–83. 10.1146/annurev.immunol.021908.132532 19105661

[96] Mosser DM The many faces of macrophage activation. J Leukoc Biol (2003) 73(2):209–12. 10.1189/jlb.0602325 12554797

[97] Mosser DM Edwards JP. Exploring the full spectrum of macrophage activation. Nat Rev Immunol (2008) 8(12):958–69. 10.1038/nri2448 19029990 PMC2724991

